# Protein kinase F regulates the virulence of *Mycobacterium tuberculosis*

**DOI:** 10.1128/iai.00698-25

**Published:** 2026-07-27

**Authors:** Flor Torres-Juarez, Shivangi Rastogi, David Young, Ashton T. Belew, Paul J. Baker, Viplov K. Biswas, Shannon E. Khan, Najib M. El-Sayed, D. Branch Moody, Katrin D. Mayer-Barber, Volker Briken

**Affiliations:** 1 Inflammation and Innate Immunity Section, Laboratory of Clinical Immunology and Microbiology, National Institute of Allergy and Infectious Diseases 35037, Bethesda, Maryland, USA; 2 Department of Cell Biology and Molecular Genetics, University of Maryland 1068 , College Park, Maryland, USA; 3 Division of Rheumatology, Immunity and Inflammation, Brigham and Women’s Hospital, Harvard Medical School 1811 , Boston, Massachusetts, USA; 4 Center for Bioinformatics and Computational Biology, University of Maryland Institute for Advanced Computer Studies, University of Maryland 1068 , College Park, Maryland, USA; 5 Center for Bioinformatics, University of Maryland Institute for Health Computing, North Bethesda, Maryland, USA; Rutgers New Jersey Medical School, Newark, New Jersey, USA

**Keywords:** tuberculosis, inflammasome, PDIM, serine/threonine kinases, virulence gene

## Abstract

The serine/threonine protein kinase F (PknF) of *Mycobacterium tuberculosis* (Mtb) has poorly defined targets and functions but is involved in limiting NLRP3 inflammasome activation in murine macrophages and dendritic cells *in vitro*. The importance of PknF for the virulence of Mtb *in vivo* is not known. Here, we demonstrate that the Mtb CDC1551 deletion mutant of *pknF* (∆*pknF*) expresses significantly increased levels of the lipid pthiocerol dimycoserosate (PDIM), a polyketide lipid with pro-virulence properties. The ∆*pknF* mutant strain compared with the Mtb and complemented strains showed a 100-fold increase in growth at day 28 and about a 10-fold increase in growth at days 90–98 in the lungs of mice. The increase in pulmonary bacterial loads after infection with the ∆*pknF* strain was conserved even in *Nlrp3*-deficient mice, arguing that PknF modulates Mtb virulence independently of NLRP3-inflammasome activation in mice. Staining of lung sections revealed increased inflammation in the lungs of ∆*pknF* strain-infected mice when compared with Mtb and the complemented mutant strain. Highly susceptible B6.Sst1^S^ mice displayed decreased host resistance, with significantly decreased survival when infected with the ∆*pknF* strain compared to the complemented strain or Mtb. In conclusion, our data suggest that expression of PknF, as a modulator of multiple downstream effector proteins, restricts the virulence of Mtb in the lungs of mice through an NLRP3 inflammasome-independent mechanism, but potentially via suppressing production of the virulence lipid, PDIM.

## INTRODUCTION

Modulation of innate immune activation after infection of host cells is a key virulence mechanism employed by intracellular bacterial pathogens, especially infection with *Mycobacterium tuberculosis* (Mtb). Here, we study the role of the Mtb serine/threonine protein kinase F (PknF), based on *in vitro* data showing its role in the regulation of the NLRP3 inflammasome activation, which tightly regulates the production of cleaved, bioactive IL-1β (1, 2). IL-1β is an important innate cytokine critical for host resistance to Mtb (3, 4). Mice deficient in the expression of IL-1α, IL-1β, or the IL-1β receptor (IL1R1) are hyper-susceptible to Mtb infections (3, 5–10). IL-1R1 confers host resistance in mice via *trans*-protection of infected cells by both immune and non-immune cells and not via infected cell-autonomous anti-microbial mechanisms (11). Although the precise mechanisms remain unclear*, in vivo*, a major function of IL-1β seems to be to induce host-protective eicosanoids and to inhibit detrimental type I interferon responses to promote cooperative tissue immunity (5, 10). Nucleotide-binding and oligomerization domain-like receptors (NLRs) survey the host cell cytosol for the presence of pathogens, and different NLRs recognize various pathogen-derived components or host cell stress to then initiate formation of the inflammasome complex (12, 13). For example, NLRP3 is activated by intracellular stress, and notably, the efflux of potassium ions (K^+^) is a major trigger (14, 15). The NLRP3 inflammasome in human and murine macrophages is slightly activated during *in vitro* infections with Mtb (16–19) via a mechanism that involves NLRP11 and caspase-4/5 in human cells (20). *In vivo*, using the mouse model, the inflammasome complex is not involved in generating IL-1β in response to Mtb infections (3, 16, 17). This inflammasome-independent IL-1β production in the mouse could potentially be due to the capacity of Mtb to inhibit the NLRP3 and AIM2 inflammasome activation (21–24). Indeed, an Mtb deletion mutant of the *ptpB* gene, which is involved in inflammasome inhibition, showed an increase in IL-1β response in the lungs of infected mice and was attenuated for growth (23).

A genetic screen revealed that *pknF* is important for inhibition of the NLRP3 inflammasome in murine macrophages (1) and dendritic cells (2) during Mtb *in vitro* infections. PknF is one of 11 members of a family of serine/threonine kinases in Mtb (25, 26). These protein kinases serve broader regulatory roles in Mtb by phosphorylating downstream target proteins that have shared function, so that the parallel phosphorylation of multiple effector programs can globally regulate Mtb physiology (25–27). The 476 amino acid (aa) PknF protein has one predicted transmembrane domain and a cytosolic 260 aa domain that has high homology with serine/threonine kinase domains of eukaryotic proteins (26). Global O-phosphorylation analysis after deletion of *pknF* revealed reduced phosphorylation on 17 sites, suggesting that they are direct targets of PknF (27). *In vitro* assays show that PknF may phosphorylate Rv1747, Rv0020C, KasB, HupB, and GroEL1 (28–31). *rv1747* is in an operon with *pknF* (*rv1746*), and the phosphorylation of the T150 and T208 residues of Rv1747 activates this presumptive ATP-binding cassette (ABC) transporter (32). Rv1747 is negatively regulated by Rv2623 (33), which is phosphorylated by PknF (27). Interestingly, the deletion of *rv1747* produces an attenuation phenotype *in vivo* (32, 34), whereas the deletion of the negative regulator *rv2623* results in hypervirulence (35). There is limited information on the importance of PknF for the virulence of Mtb *in vivo* (36). In the present study, we investigated how deletion of *pknF* affects the virulence of the bacteria in both susceptible and resistant mouse strains and specifically tested for connections with NLRP3 inflammasome function based on our previous *in vitro* findings.

## RESULTS

**Fig 1:**
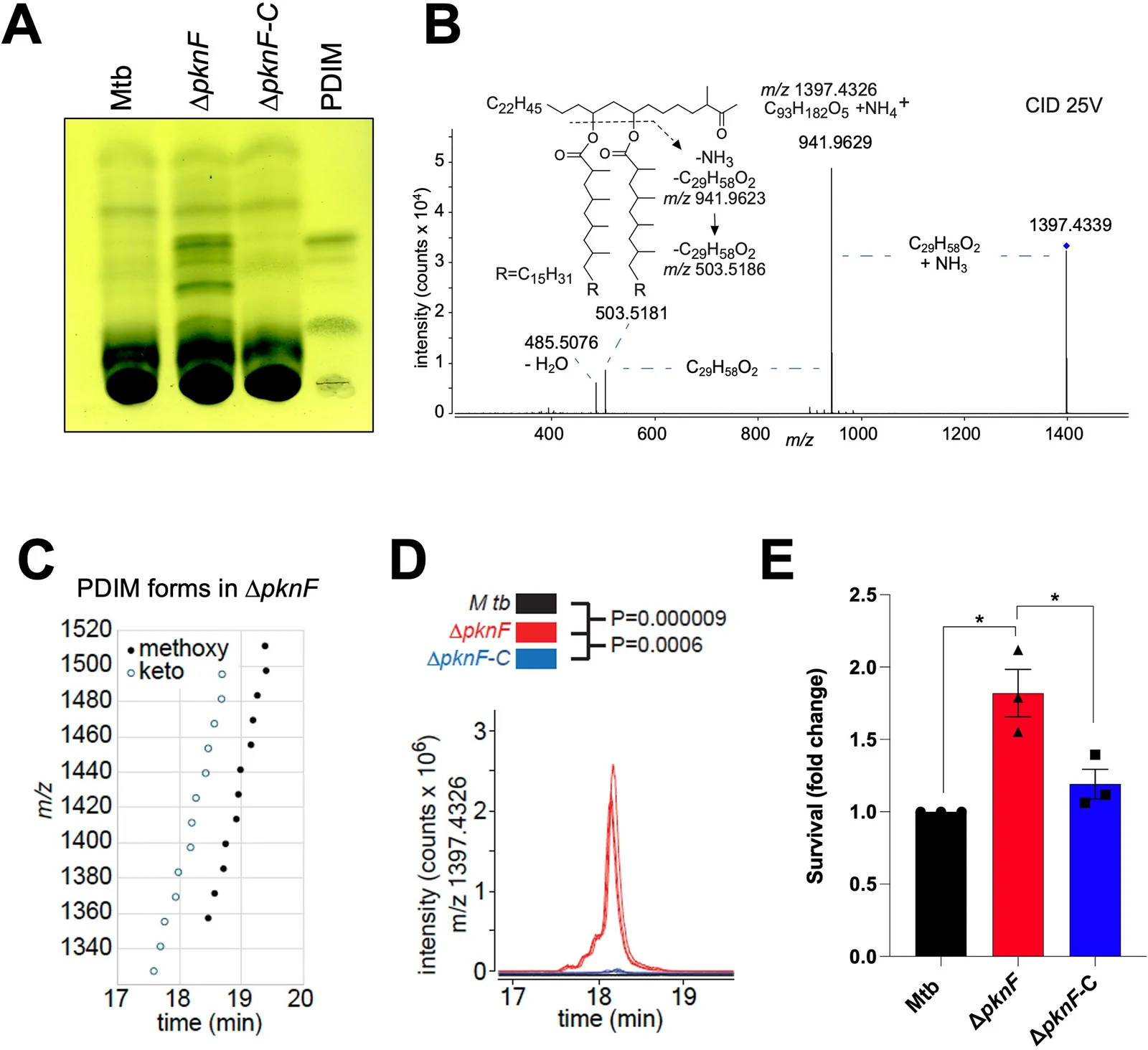
The deletion of *pknF* in Mtb CDC1551 causes an increase in PDIM expression. (**A**) Total lipids extracted from the indicated wild-type Mtb (CDC1551), the *pknF* deletion mutant (∆*pknF*), and complemented mutant strains (∆*pknF*-C), along with purified PDIM, were separated by thin-layer chromatography. A representative result of three independent lipid extractions is shown. (**B**) The structure of PDIM, which is deduced at the keto form along with two C29 mycocerosic acid moieties based on ESI-CID MS of ammonium adducts in the positive mode. (**C**) Fulfilling the expected biological distributions with chain length analogs that appear in alkane series, deduced methoxy- and keto-PDIM forms were upregulated in the ∆*pknF* strain. (**D**) Mass spectral intensity for C93 keto-PDIM detected in mass-normalized lipid extracts from wild-type Mtb (black), ∆*pknF* (red), and ∆*pknF*-C (blue) is shown in biological triplicate. (**E**) The indicated bacterial strains were grown in the presence or absence of vancomycin for 10 days, and their relative growth was measured via optical density at 600 nm. The % growth was normalized to Mtb and plotted as a fold change in survival. Statistical analysis was performed via one-way ANOVA (**B**) or negative binomial (**E**) (*P < 0.05, **P < 0.01, ***P < 0.001, ****P < 0.0001).

The continuous culture of Mtb in liquid medium often results in the loss of pthiocerol dimycocerosate (PDIM) (37, 38). PDIM is an important driver of Mtb virulence in the mouse model (39–41). Consequently, we analyzed PDIM levels in our CDC1551 wild-type Mtb, ∆*pknF* mutant, and complemented mutant ∆*pknF*-C strains by performing an extraction of total lipids and separating multiple PDIM molecular species on a thin-layer chromatography plate (Fig. 1A). Interestingly, the ∆*pknF* strain showed increased PDIM production when compared to the wild-type strain and, importantly, the complemented mutant strain ∆*pknF*-C (Fig. 1A). These results demonstrated a complementable phenotype of increased PDIM biosynthesis in the *pknF* deletion mutant.

Whereas TLC identification relies on interpretations of banding patterns and comigration with standards, mass spectrometry (MS) analysis can provide direct detection of molecules based on their accurate mass, which allows calculation of their chemical formulas, and, with collision-induced dissociation (CID), detection of chemical components of macromolecules for direct and unambiguous chemical assignment of the identity and substructure of molecules detected. Positive mode electrospray ionization (ESI) time of flight (TOF) MS detected an ion of *m/z* 1397.433 that matched the ammonium adduct of PDIM ([M + NH3], C93H18205). CID-MS detected neutral loss fragments corresponding to the loss of one (*m*/*z* 941.962) or two (m/z 503.519) C29 mycocerosate chains, allowing a deduced assignment of a C35 keto-modified backbone (C93 keto-PDIM) (Fig. 1B). Further confirming the match with the expected patterns of a natural Mtb-produced PDIM lipid family, we also detected a mass matching the ammonium adduct of a C93 methoxy PDIM (Fig. 1C) and its expected alkyforms. For example, we detected two alkane series, corresponding to the expected number and overall size of the two forms of PDIM comprised of methoxy and keto backbones. We further detected 12 chain length analogs in two alkane series, where the median (C94) and range (C88–101) of length match the reported length of PDIM produced by Mtb when grown in culture (42, 43). Finally, the spectral intensity counts in high-performance liquid chromatography-MS analysis of a representative C93 keto-PDIM for Mtb (black), ∆*pknF* (red), and ∆*pknF*-C (blue) derived from three independent total lipid extractions showed that PDIM signals were significantly elevated in the ∆*pknF* mutant when compared to the wild-type strain, and complementation reversed the phenotype (Fig. 1D). A quantitative analysis of PDIM at *m*/*z* 1497.5578 was carried out using the method of standard additions and a standard of PDIM A synthesized at that mass. We found a PDIM concentration for ∆*pknF* was 3.5 μM, for wild-type CDC1551 0.22 μM, and 0.13 μM for the complemented mutant strain (Fig. S1). This result, using a gold standard quantification that documents a 16-fold change in PDIM quantity after gene deletion, is in agreement with other qualitative measures presented in Fig. 1D. In addition, we performed a vancomycin resistance assay, since PDIM levels positively correlate with vancomycin resistance (38). All three strains are susceptible to vancomycin and show a reduced total growth rate when compared to no vancomycin treatment. Nevertheless, the ∆*pknF* strain exhibited an almost twofold increase in vancomycin resistance relative to wild-type or ∆*pknF*-C strains (Fig. 1E). Next, we investigated the potential for selective point mutations in the PDIM operon via whole-genome sequencing of all three strains (see Materials and Methods). We found a point mutation in the coding sequence of the *ppsB* gene in all three strains at position 3,251,028 (“G” to “A” change), which did not result in a change in amino acid. Another mutation in the *ppsB* gene could only be detected in the wild-type strain (position 3,248,288 “C” to “A” change, leading to “P” to “Q” amino acid change) and hence cannot explain the low levels of PDIM in the complemented strain. Taken together, our results demonstrate that the deletion of *pknF* leads to a significant increase in PDIM, identifying PknF as a negative regulator of PDIM pools in Mtb CDC1551.

**Fig 2:**
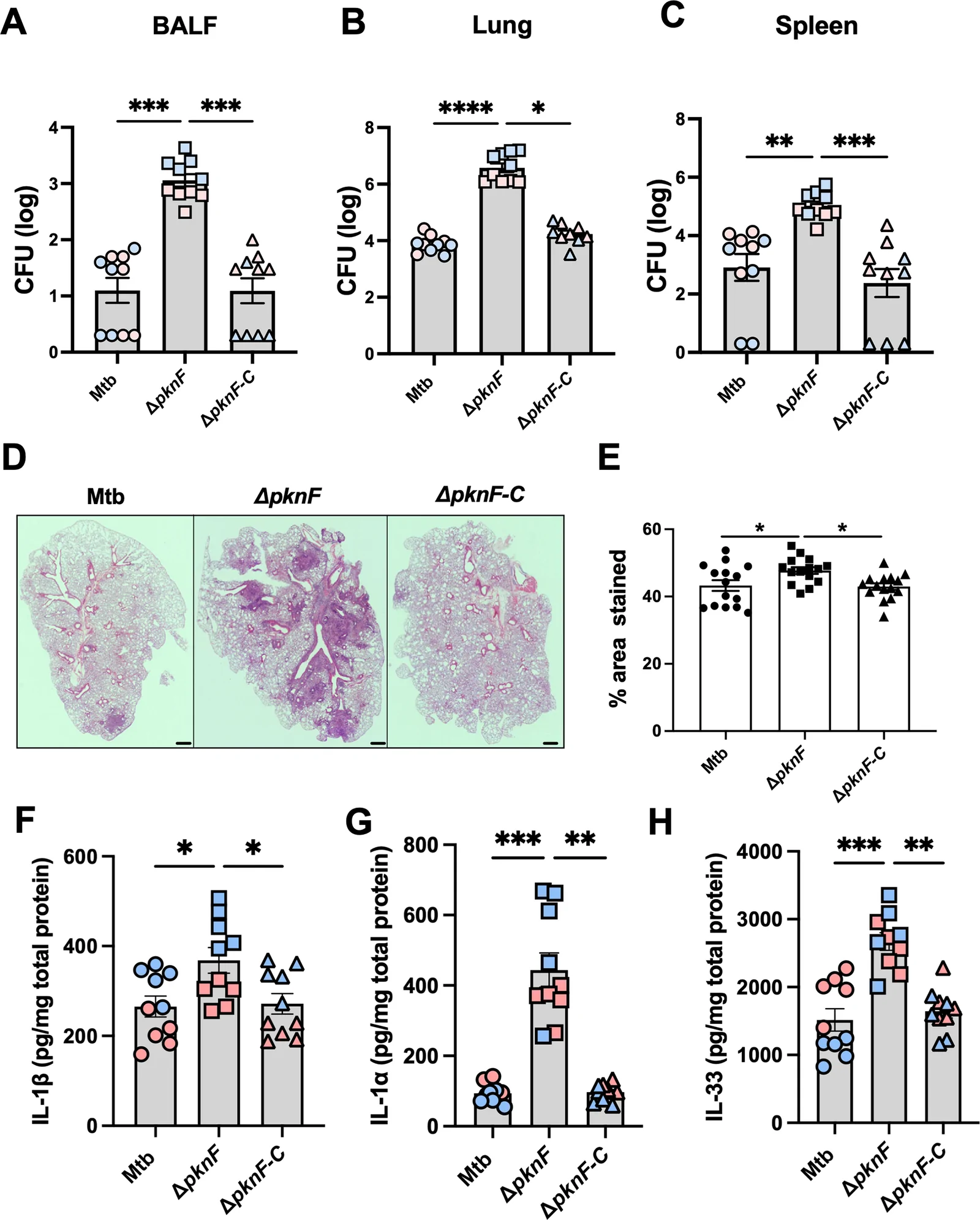
The deletion of *pknF* causes an increase in the virulence of Mtb in C57Bl/6 mice. Mice were infected with 100–200 CFU (day 1 post-infection CFU, not shown) of the indicated bacterial strains via intrapharyngeal delivery. After 28 days, the CFU in the (**A**) BALF, (**B**) lungs, and (**C**) spleen were determined. Each point represents one infected mouse from two independent infections (blue and pink colors), the dashed line is the limit of detection. (**D and E**) Three slices per lung from five different mice were stained with hematoxylin and eosin (black line is scale bar showing 300 μm). Image quantification was done via ImageJ. (**F–H**) Multiplexed ELISA (Luminex) was used to determine the amount of indicated cytokine in the lungs, normalized to total protein. Normality of each data set was tested. Kruskal-Wallis with Dunn’s post-test (**A–C**) and one-way ANOVA with Tukey’s post-test (**D–H**) were performed to compare experimental groups (*P < 0.05, **P < 0.01, ***P < 0.001, ****P < 0.0001).

Given the large fold-change in PDIM pools (Fig. 1D; Fig. S1), we hypothesized that the observed increase in PDIM of the ∆*pknF* strain would correspond to increased virulence in mice after Mtb infection, due to the known role of PDIM in promoting *in vivo* virulence (39–41). To test this hypothesis, we infected C57Bl/6 mice with 100–200 CFU (day 1 post-infection CFU, not shown) of the indicated bacterial strains via intrapharyngeal pulmonary delivery. After 28 days, the CFU in the bronchoalveolar lavage fluid (BALF) (Fig. 2A), lungs (Fig. 2B), and spleen (Fig. 2C) were 100-fold increased in mice infected with the ∆*pknF* strain compared to Mtb or the complemented strain. The increase in CFU corresponded with significantly increased inflammation as assessed by hematoxylin and eosin staining of lung sections (Fig. 2D and E). To further assess changes in host responses, we measured inflammatory cytokines in the lungs of mice infected with Mtb, ∆*pknF* strain, or the complemented mutant strain (Fig. 2F through H; Fig. S1). The proinflammatory cytokines MCP-1, CXCL10, GM-CSF, IFNγ, and TNFα all showed a significant increase in ∆*pknF* mutant-infected mice when compared to wild-type and ∆*pknF*-C-infected lungs (Fig. S2A through E), while IL-4, IL-6, IL-10, IL12p70a, and IL-17 showed no statistically significant differences (Fig. S2F through J). Similarly, in addition to IL-1β (Fig. 2F), the IL-1 family members IL-1α (Fig. 2G) and IL-33 (Fig. 2H) were both significantly elevated in the lungs of ∆*pknF*-infected mice, likely correlating with increased tissue necrosis and bacterial burden.

**Fig 3:**
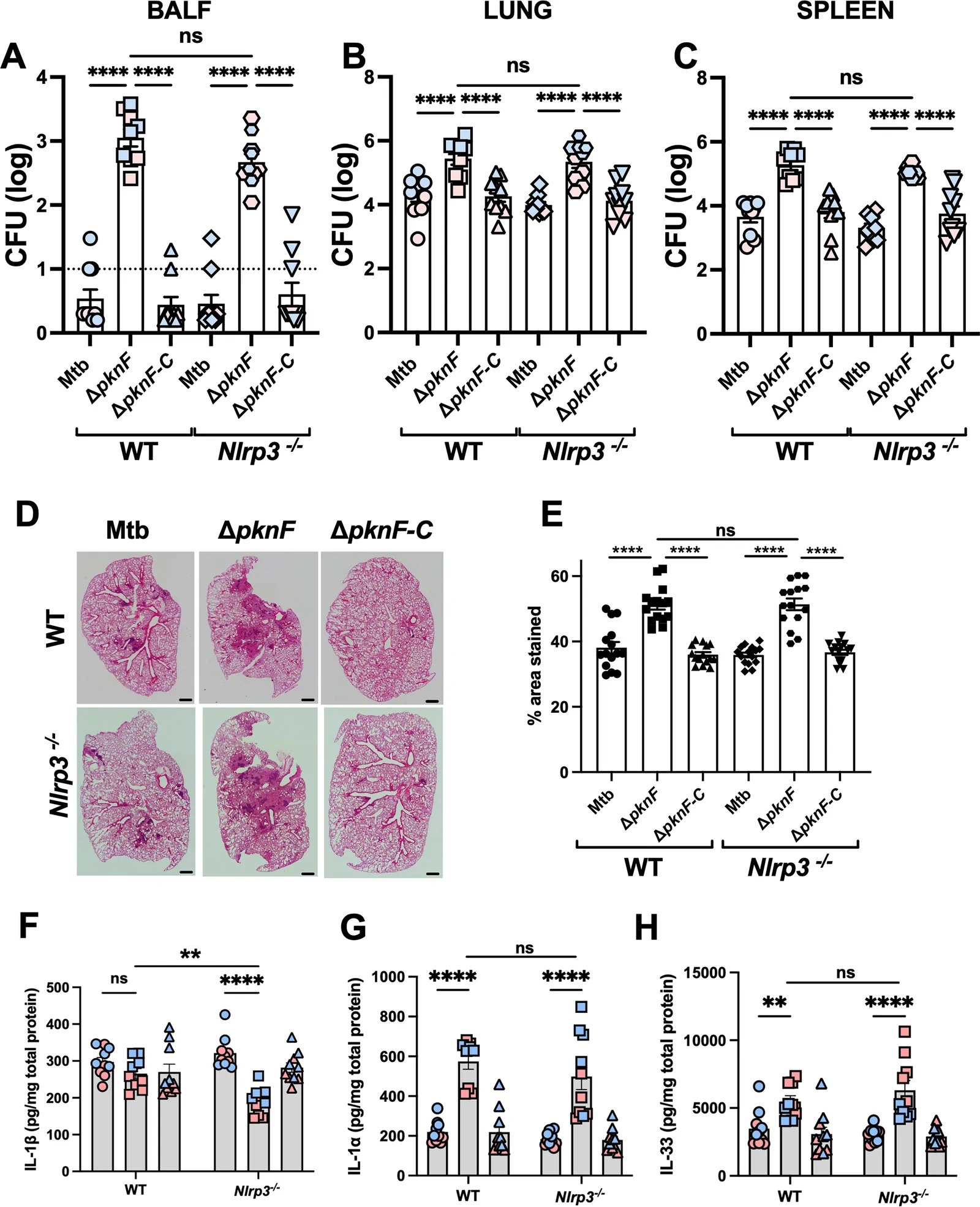
The increased virulence of the Mtb∆*pknF* mutant is independent of NLRP3. C57Bl/6 mice (WT) and *Nlrp3* knock-out mice (*Nlrp3^−/−^*) were infected with 100–200 CFU of the indicated bacterial strains via intrapharyngeal delivery. After 90–98 days, the CFU in the (**A**) BALF, (**B**) lungs, and (**C**) spleen were determined. Each point represents the result of one infected mouse from two independent infections (blue and pink colors); the dashed line is the limit of detection. (**D and E**) Three slices per lung from five different mice were stained with hematoxylin and eosin (black line is scale bar showing 350 μm). Image quantification was done via ImageJ. (**F–H**) Multiplexed ELISA (Luminex) was used to determine the amount of indicated cytokine in the lungs, normalized to total protein. Circles indicate wild-type Mtb, squares the ∆*pknF* strain, and triangles the complemented ∆*pknF* strain. Normality of each data set was tested, and two-way ANOVA with Tukey’s post-test was performed to compare experimental groups (*P < 0.05, **P < 0.01, ***P < 0.001, ****P < 0.0001; ns, not significant).

To experimentally test whether the detected increase in bacterial burden and IL-1β in the lungs of mice infected with ∆*pknF* was due to the lack of the ability to suppress NLRP3 inflammasomes as shown before *in vitro* (1), we next infected *Nlrp3* knock-out mice (*Nlrp3*^−/−^) or C57Bl/6 mice (WT B6) with 100–200 CFU (day 1 post-infection CFU, not shown). Similar to our observations after 28 days, 3 months after infection, the BALF (Fig. 3A) exhibited a 100-fold increase for ∆*pknF* bacteria compared to Mtb and ∆*pknF*-C. This increase was between 10-fold and 50-fold in the lungs (Fig. 3B) and spleen (Fig. 3C). Importantly, deletion of *Nlrp3* did not significantly change the amount of ∆*pknF* bacteria in the BALF, lungs, or spleen (Fig. 3A through C), arguing that dysregulated NLRP3 activation alone was unlikely to cause the loss of bacterial control in mice infected with ∆*pknF* Mtb. In line with our observations above, hematoxylin and eosin staining revealed increased lung inflammation and involvement in the ∆*pknF* mutant-infected mice compared to the wild-type Mtb and ∆*pknF*-C, which was unaffected by the deletion of *Nlrp3* (Fig. 3D and E). Despite increased bacterial loads at 3 months after infection in the lungs of mice infected with ∆*pknF* compared to Mtb and ∆*pknF*-C, we observed no changes in IL-1β levels in the lungs of WT B6 mice infected with ∆*pknF* compared to Mtb or d ∆*pknF*-C (Fig. 3F), while the alarmins IL-1α (Fig. 3G) and IL-33 (Fig. 3H) remained elevated. However, while bacterial burden was similarly increased in ∆*pknF*-infected *Nlrp3^−/−^* mice compared to Mtb and ∆*pknF*-C, IL-1β protein in the lungs of ∆*pknF*-infected *Nlrp3^−/−^* mice was significantly reduced compared to Mtb and ∆*pknF*-C-infected *Nlrp3^−/−^* mice (Fig. 3F). These results suggest that while the control of bacterial replication via PknF may occur independently of NLRP3, the modulation of IL-1β production via PknF required NLRP3 *in vivo,* as previously documented *in vitro* using bone marrow-derived macrophages (1) and dendritic cells (2). In contrast, protein levels of IL-1α (Fig. 3G) and IL-33 (Fig. 3H) were increased in ∆pknF Mtb-infected *Nlrp3^−/−^* lungs compared to lungs from *Nlrp3^−/−^* mice infected with Mtb or ∆*pknF*-C. No differences were observed in MCP-1, CXCL10, GM-CSF, TNFα, IFNγ, IL-4, IL-6, IL-10, or IL-12 between ∆*pknF*-infected WT B6 or *Nlrp3^−/−^* mice (Fig. S3A through E, G through J). Of note, IL-17, an IL-1 inducible cytokine, was decreased in the lungs of ∆*pknF*-infected *Nlrp3^−/−^* mice compared to Mtb-infected *Nlrp3^−/−^* mice, but the similar trend in WT B6-infected mice was not statistically significant (Fig. S3F). In both WT B6 and *Nlrp3^−/−^* mice, levels of IL-6 and IL-12p70 were reduced in ∆*pknF*-infected lungs compared to lungs of mice infected with Mtb or ∆*pknF*-C (Fig. S3H and J).

**Fig 4:**
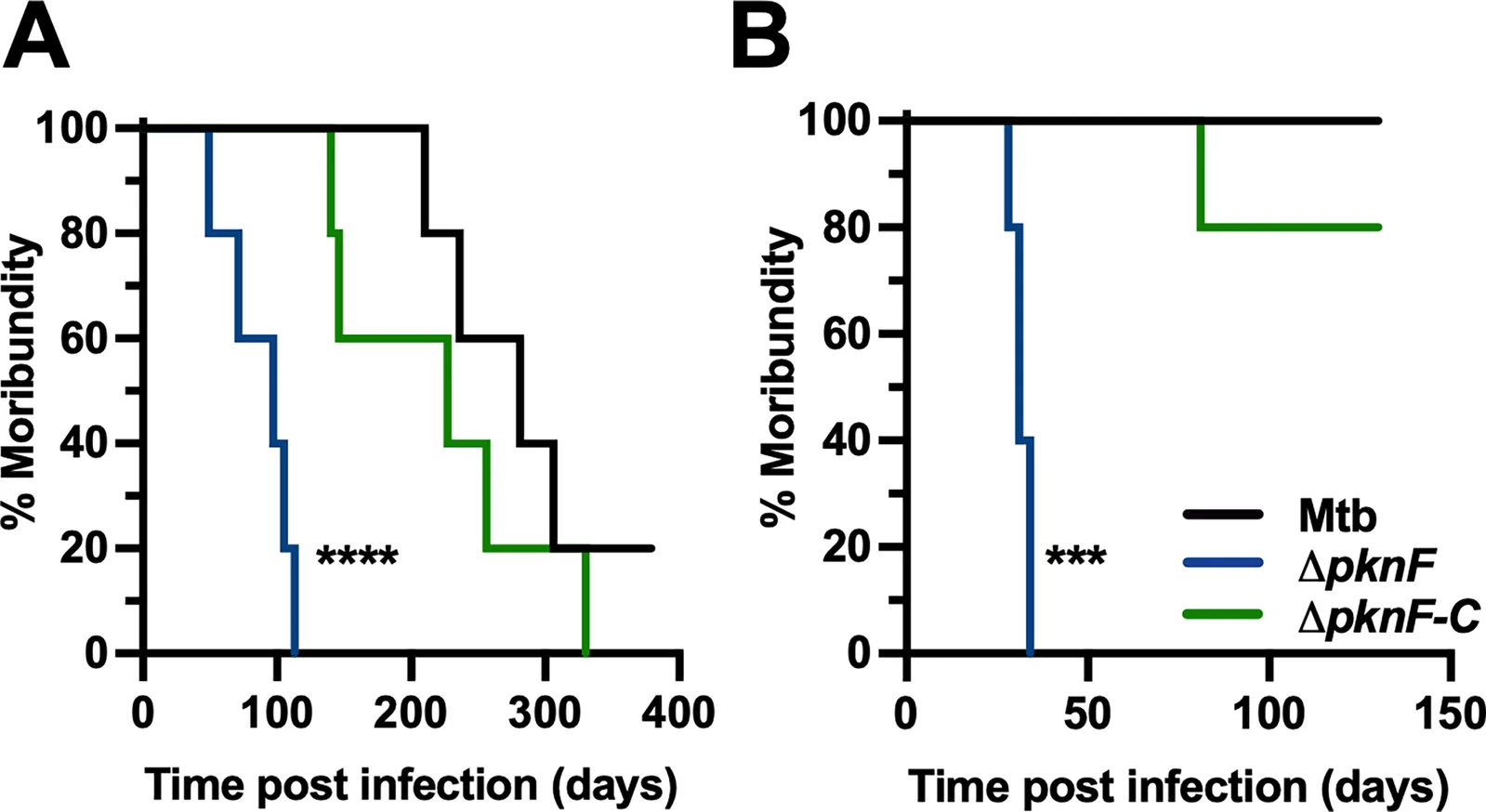
Enhanced virulence and reduced host resistance of the Mtb∆*pknF* mutant in B6.Sst1^S^ mice. Mice (*n* = 5) were infected with (**A**) about 60 CFU or (**B**) about 600 CFU of Mtb (black), ∆*pknF* (blue), or a complemented ∆*pknF* strain (∆*pkn*F-C, green). The moribundity was monitored for the indicated times. The curves were compared using the log-rank Mantel-Cox test (***P < 0.001, ****P < 0.0001).

Finally, to assess the virulence of the ∆*pknF* strain in the context of lower host resistance, we utilized highly susceptible mice carrying the C3HeB/FeJ-derived sst1-susceptible (sst1^S^) locus on a B6 background (B6.Sst1^S^ mice) that develop well-organized necrotic granulomas and succumb rapidly to Mtb infection (44). Consistent with the observed increase in bacterial loads in both WT B6 and *Nlrp3^−/−^* mice, host resistance in B6.Sst1^S^ mice was reduced with significantly increased moribundity in mice infected with around 60 CFU of ∆*pknF* compared to Mtb or ∆*pknF*-C (Fig. 4A). When mice were infected with a 10-fold higher dose, the median time to moribundity of ∆*pknF*-infected mice was only 31 days (Fig. 4B) compared to 97 days with 60 CFU infection. The higher dose experiment was terminated after 130 days since none of the wild-type Mtb-infected mice had died by then, and only one of the ∆*pknF*-C-infected mice. These data support that Mtb PknF is a potent modulator of virulence and host resistance in both resistant and susceptible mice.

## DISCUSSION

We identified Mtb *pknF* in a screen for Mtb genes that mediate host cell inflammasome inhibition *in vitro* (1). We now show that the deletion of *pknF* significantly increases the levels of PDIM in the Mtb CDC1551 strain. Acting as a long-chain polyketide lipid that resides near the surface of Mtb, PDIM was one of the first genetically assigned virulence-controlling lipids (40, 41). PDIM loss occurs commonly during *in vitro* growth of Mtb (37). In the past, this has led to potential for incorrect assignment of virulence effects to certain genes just because the Mtb deletion mutant had lost its PDIM expression. It is thus notable that in our data, the complementation of *pknF* deletion reverses biochemical PDIM phenotypes as measured by TLC detection and HPLC-MS detection, as well as functional phenotypes like vancomycin response, *in vivo* immune responses, and bacterial survival.

Furthermore, complete PDIM loss due to inactivation of any of the many genes required for its biosynthesis commonly occurs during *in vitro* growth of Mtb (37), and other work showed physiological regulation across a spectrum of biosynthesis levels (38). In this setting of an emerging understanding that this crucial virulence lipid is actively tuned by Mtb, our results suggest a specific mechanism of tuning whereby expression of the serine/threonine protein kinase PknF downregulates PDIM levels, inviting future work to identify counterregulatory activators of PDIM biosynthesis in Mtb that may either act directly through PknF or act independently of this regulatory pathway. For example, the deletion of protein kinase H (*pknH*) of Mtb results in a decrease in PDIM production (45). The precise mechanisms for this decrease remain unclear. Both PknF and PknH belong to the same family of serine/threonine protein kinases, arguing that enzymes involved in the biosynthesis or degradation of PDIM are regulated by serine/threonine phosphorylation. In fact, phosphoproteomic analysis of a *∆pknF* Mtb strain showed a reduced phosphorylation of serine residue 447 in FadD28, an enzyme involved in the biosynthesis of PDIM (27). The phosphorylation of serine 499 of FadD28 is upregulated in the *∆pknH* deletion mutant, suggesting an indirect effect of PknH on the phosphorylation of this site (27). The transcriptional repressor, Rv3167c, suppresses genes in the PDIM biosynthesis and transport operon, leading to increased PDIM levels in the *∆rv3167c* Mtb mutant (46). A single-nucleotide polymorphism in the β subunit of RNA polymerase gene (*rpoB*), which is the most common cause of acquired rifampin resistance, leads to increased PDIM levels (47, 48) via transcriptional upregulation of genes involved in PDIM biosynthesis (48). The question remains as to how precisely these pathways that regulate PDIM levels are interconnected and potentially employed by Mtb during infections *in vivo*.

PDIM is a major virulence determinant of Mtb (39–41). We show here that the ∆*pknF* mutant Mtb strain, with its increased PDIM expression, exhibits increased virulence accompanied by decreased host resistance as demonstrated by increased pulmonary bacterial loads and significantly decreased survival in infected mice compared to the parental Mtb strain. These results are consistent with our previous finding that the ∆*rv3167c* Mtb strain, which has increased PDIM, was more virulent in the C57Bl/6 mouse model after aerosol infection (49). The *∆pknH* strain of Mtb, with lower PDIM expression (45), was more virulent in BALB/c mice after intravenous infections (50). This discrepancy could be due to a different route of infection or that PknH affects the virulence of Mtb in other ways, independent of PDIM synthesis. Despite all these compelling data on the importance of PDIM for virulence in the mouse model, few data are available on the molecular mechanisms underlying this phenotype *in vivo*. There are numerous studies assigning multiple functions to PDIM during Mtb’s *in vitro* interaction with macrophages (51). One of these is its capacity to limit autophagy in human macrophages (52). A genetic screen revealed that PDIM is also important for limiting autophagy-mediated killing of Mtb in murine BMDMs (53). Importantly, this study clearly delineated the importance of PDIM-mediated autophagy inhibition for the *in vivo* virulence of Mtb (53). A limitation of our study is that we do not provide definitive evidence that the high virulence of the ∆*pknF* mutant is due to its increased PDIM production. The creation of a double mutant by deleting a PDIM biosynthesis gene (e.g., ∆*ppsD*) in the background of a ∆*pknF* mutant would provide a genetic system to determine the importance of PDIM for the virulence phenotype of the ∆*pknF* mutant. In addition, the kinase activity of PknF most likely phosphorylates other targets that are not involved in PDIM synthesis, and hence, those PknF activities could also contribute to the virulence phenotype of the ∆*pknF* mutant.

Another prominent PDIM function is its role in permeabilizing the phagosomal membrane and allowing for escape of the bacteria from the phagosome (46, 54–57). The escape of Mtb from the phagosome has been correlated with its capacity to induce cell death (46, 49, 58, 59). The high PDIM level Mtb mutant ∆*rv3167c* increased host cell necrosis via an uncharacterized cell death pathway (49). We previously demonstrated that in BMDMs and BMDCs, the ∆*pknF* mutant induces an increase in pyroptosis by activating the NLRP3-inflammasome (1, 2). Our data suggest that *in vivo*, pyroptosis is not a major cell death pathway induced by ∆*pknF*, since the increased virulence and bacterial burdens of the mutant are similar between WT B6 and *Nlrp3^−/−^* mice. Nevertheless, and consistent with our initial *in vitro* observations (1), Nlrp3 inflammasome-dependent IL-1β production remained PknF dependent in the lungs of mice, since *Nlrp3^−/−^* mice infected with ∆*pknF* had significantly lower levels of IL-1β in the lungs than *Nlrp3^−/−^* mice infected with Mtb or ∆*pknF*-C.

Overall, our data reveal a previously unrecognized role for PknF in the modulation of bacterial virulence *in vivo*. In addition, our results point toward new directions for mechanistic studies examining the role of PknF in Mtb PDIM synthesis modulation.

## MATERIALS AND METHODS

### Mice

C57BL/6NTAC mice were obtained from Taconic Bioscience (NY, USA). C57BL/6 mice expressing a Foxp3-GFP reporter (C57BL/6-Foxp3^tm1Kuch^ [60]) or Thy1.1 (B6.PL-*Thy1^a^*/CyJ) were also used as wild-type (WT B6) C57BL/6 controls with similar results to C57BL/6NTAC mice. C57BL/6-Foxp3^tm1Kuch^, B6.PL-*Thy1^a^*/CyJ, *Nlrp3^−/−^* (B6N.129-*Nlrp3^tm2Hhf^*/J [61]), and B6 Sst1 (B6J.C3-sst1^C3HeB/Fej^ [44]) were obtained through a supply contract between NIAID and Taconic Farms. Mice of both sexes were used and 12–18 weeks old at the onset of experiments. Mice within experiments were age and sex matched.

### Mtb infection of mice

Mtb CDC1551 WT, *pknF* deletion mutant (MtbΔ*pknF*), and *pknF* complemented (Δ*pknF*-C) strains (1) were grown in Middlebrook 7H9 broth (Sigma Aldrich, Darmstadt, Germany) supplemented with 10% OADC, 0.5% glycerol, and 0.05% Tween-80; for mutant and complement strains, the antibiotics hygromycin (50 µg/mL) and kanamycin (50 µg/mL) were added, respectively. Bacterial concentration in a log phase was determined by OD_600_. Briefly, once bacteria reached OD = 0.6, bacteria were centrifuged at 5,000 rpm for 10 min. Then, resuspended in PBS and flushed through a 27-gauge needle. Finally, the bacteria were diluted in PBS targeting 100–200 CFU per 50 µL of inoculum per mouse. The bacteria were delivered to the lungs of mice via intrapharyngeal (i.ph.) route, and infection doses were confirmed by plating lung homogenates 1 day post-infection. For survival studies, mice were monitored twice daily by NIAID animal facility staff for signs of moribundity (severely hunched posture, vertebrae distinctly segmented, labored breathing, wobbling gait, and immobility) and humanely euthanized when they presented two or more signs.

### CFU determination

Lung and spleen homogenates were generated using the Precellys Evolution machine (Bertin Instruments, France). Serial dilutions of homogenized tissue and BALF were done in PBS 0.05% Tween 20, then plated on Middlebrook H11 agar plates (Sigma Aldrich, Darmstadt, Germany) supplemented with 10% OADC (Oleic Acid-Albumin-Dextrose-Catalase; Beckton Dickinson, MD, USA) and 0.5% glycerol (Invitrogen, Karnataka, India). CFUs were counted after 21 days of incubation at 37°C.

### Lung cytokine/chemokine quantification and histology

Lung homogenates were generated using the Precellys Evolution machine (Bertin Instruments, France). Samples were centrifuged and sterile-filtered using a 0.22 µm filter and then stored at −80°C. The cytokine/chemokine analysis was done by using the Luminex MAGPIX platform according to the manufacturer’s protocol (R&D Bioscience). BCA assays were performed on lung homogenates to normalize cytokine levels as pg per mg of total protein.

The right lower lobes of the lungs were fixed in 10% buffered formalin for histopathology. Lung lobes were washed and suspended in PBS, followed by paraffin embedding, sectioning, and hematoxylin and eosin (H&E) staining done by Histoserv Inc (https://www.histoservinc.com/). The tile scanning and image stitching were performed for each mouse lung section, and slides were imaged on a Nikon Eclipse microscope. The images were generated using Nikon’s NIS-Elements imaging software. Quantification of H&E-stained lung sections was done using ImageJ software. Paraffin embedding, sectioning, and hematoxylin and eosin staining were done by Histoserve Inc (https://www.histoservinc.com/).

### Lipid analysis

Lipid extracts were created as described (42) and analyzed using an Agilent 6546 Q-Tof mass spectrometer connected to an Agilent Infinity II liquid chromatography system using normal-phase chromatography was performed as described (42). Total lipid extracts of WT Mtb, ∆pknF, and ∆pknF-C strains were analyzed for PDIM levels using one-dimensional TLC. PDIMs were developed using petroleum ether:diethyl ether (9:1), and lipid spots were visualized by staining with 5% solution of molybdophosphoric acid in 95% ethanol, followed by charring at 140°C.

Reversed phase chromatography was done on an LC-MS system using an Agilent Poroshell 120 column (EC-C18, 1.9 mm 3 × 50 mm) with gradient elution. Mobile phase A was 5:95 water:methanol, and mobile phase B was 85:15 n-propanol:cyclohexane. The gradient started at 100% A, from 2 min to 15 min, it increased to 100% B, holding at 100% until 23 min. It then returned to 100% A and held from 25–30 min to recondition the column. The flow rate was 0.15 mL/min throughout the run. Collisionally induced dissociation was done using a narrow (1.3 *m/z*) isolation window and 25 V collision energy.

Quantitative analysis of PDIM at *m*/*z* 1497.5578 was carried out using the method of standard additions and a standard of PDIM A synthesized at that mass (62). The PDIM standard was kindly provided by Dr. Adriaan Minnaard (Groningen University), and 10 µL of lipid extract (1.0 mg/mL) was combined with 10 µL of standard in the starting mobile phase at different concentrations. 20 µL was then injected for LC-MS analysis. Linear regression (of the peak areas in the mass chromatogram at *m/z* 1497.5578) was used to extrapolate to the X-axis and estimate the original concentration. Reversed phase chromatography for PDIM quantification was done using the same solvents and program, except that the gradient used 50% B as the starting mobile phase to improve dissolution of lipophilic PDIMs.

To assess PDIM levels via a functional assay, we performed a vancomycin resistance assay as described previously (38). Briefly, assays were performed in 96-well plates in 7H9/glycerol/OADC/0.05% tyloxapol medium supplemented with 0.1 mM propionate ± 10 µg/mL of vancomycin (VAN10 vs VAN0). Bacteria were incubated at an initial OD_600_ of 0.005, and growth was measured after 10 days. The relative growth was calculated as follows: (VAN10 OD/VAN0 OD) × 100 = VAN10 %growth (38). Since there was variability in the percent growth between experiments but the relative differences between the strains remained constant, we normalized the VAN10 %growth for each strain to the growth detected in the wild-type CDC1551 strain.

### Genome sequencing

The genome sequence of CDC1551 strain of *Mycobacterium tuberculosis* was downloaded from the NCBI (GCF_000669715.1) as a reference, along with the wild-type, ∆pknF knockout, and complement mutant strains genome sequence medium-length read assemblies produced by plasmidsaurus (https://plasmidsaurus.com/). These were used as input by the progressive alignment method in mauve (63) and the default method of parsnp (64) with options to use the CDC1551 reference genbank file, output a vcf table, and use muscle (65) for the sequence alignments. The resulting alignments were visually inspected in mauve and gingr; the harvest vcf file was read into R merged against the set of CDS annotations provided by the reference assembly genbank file.

### Statistical analyses

Statistical analysis was performed using GraphPad Prism version 10.0 software. Data were analyzed using Kruskal-Wallis with Dunn’s post-test. One-way or two-way ANOVA with Tukey post-test was performed as indicated after normal distribution was confirmed using the Shapiro-Wilk test and QQ plot analysis. Time to moribundity studies were assessed using the log-rank Mantel-Cox test for survival. *P*-value significance is as follows: *- ≤ 0.05, ** - ≤ 0.01, *** - ≤ 0.001, and **** - 0.0001.

## Data Availability

The original genome sequencing data are deposited in NCBI under BioProject number PRJNA1433802.

## SUPPLEMENTAL MATERIAL

Supplemental figures iai.00698-25-s0001.pdf Fig. S1 to S3.

ASM does not own the copyrights to Supplemental Material that may be linked to, or accessed through, an article. The authors have granted ASM a non-exclusive, world-wide license to publish the Supplemental Material files. Please contact the corresponding author directly for reuse.
