# Supplementary material for: Protein kinase F regulates the virulence of Mycobacterium tuberculosis
Source: Infect Immun. 2026 Jul 27;94(9):e00698-25. doi: 10.1128/iai.00698-25 (PMC13552113; doi:10.1128/iai.00698-25)
Supplement: Supplementary file 1 [file iai.00698-25-s0001.pdf]

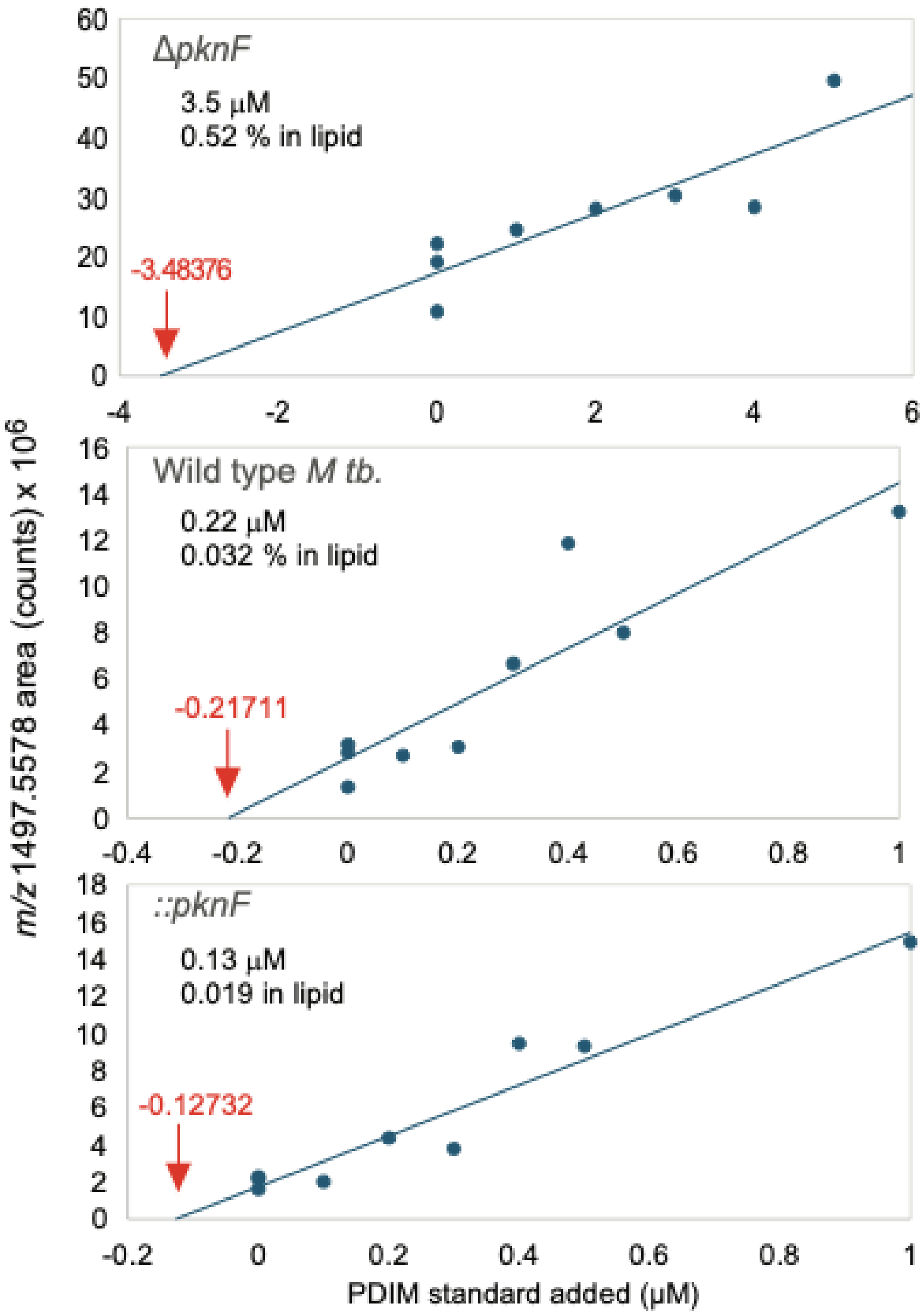

Supp. Fig. 1: Quantification of phthiocerol dimycocerosate. Synthetic standard for the methoxy form of PDIM ($C_{100}H_{198}O_5$) was used to estimate PDIM levels using the method of standard addition. Lipid extracts from (A) Δ*pknF*, (B) Wild type *M tb*. and (C) the complemented mutants strains (*::pknF)* dissolved in starting mobile phase were mixed with an equal volume of the same mobile phase containing differing concentrations of PDIM standard. Extrapolation to the X-axis yielded an estimate of the concentration in the original *M tb*. lipid solution which was at 1.0 mg/mL total lipid.

SF 2

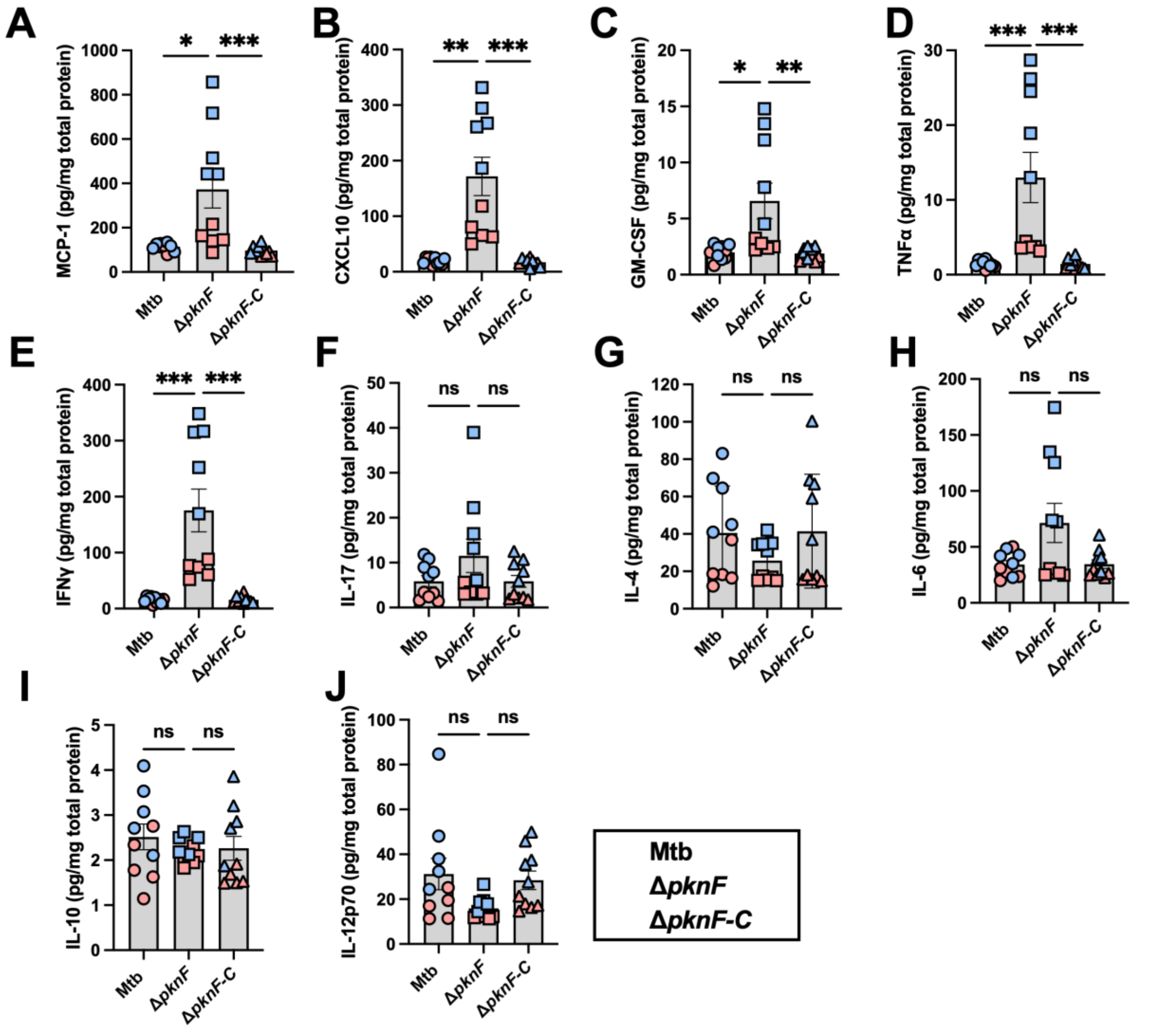

Supp. Fig. 2: Characterization of cytokine levels in lungs at day 28 after Mtb infection. Multiplexed ELISA (Luminex) was used to determine the amount of indicated cytokine in the lungs normalized to total protein. Each point represents one infected mouse from two independent infections (blue and pink colors). Normality of each data set was tested. (A-E, G, H) Kruskal-Wallis with Dunn's post-test and (F, I, J) one-way ANOVA with Tukey's post-test were performed to compare experimental groups (* $p<0.05$, ** $p<0.01$, *** $p<0.001$, **** $p<0.0001$).

SF 3

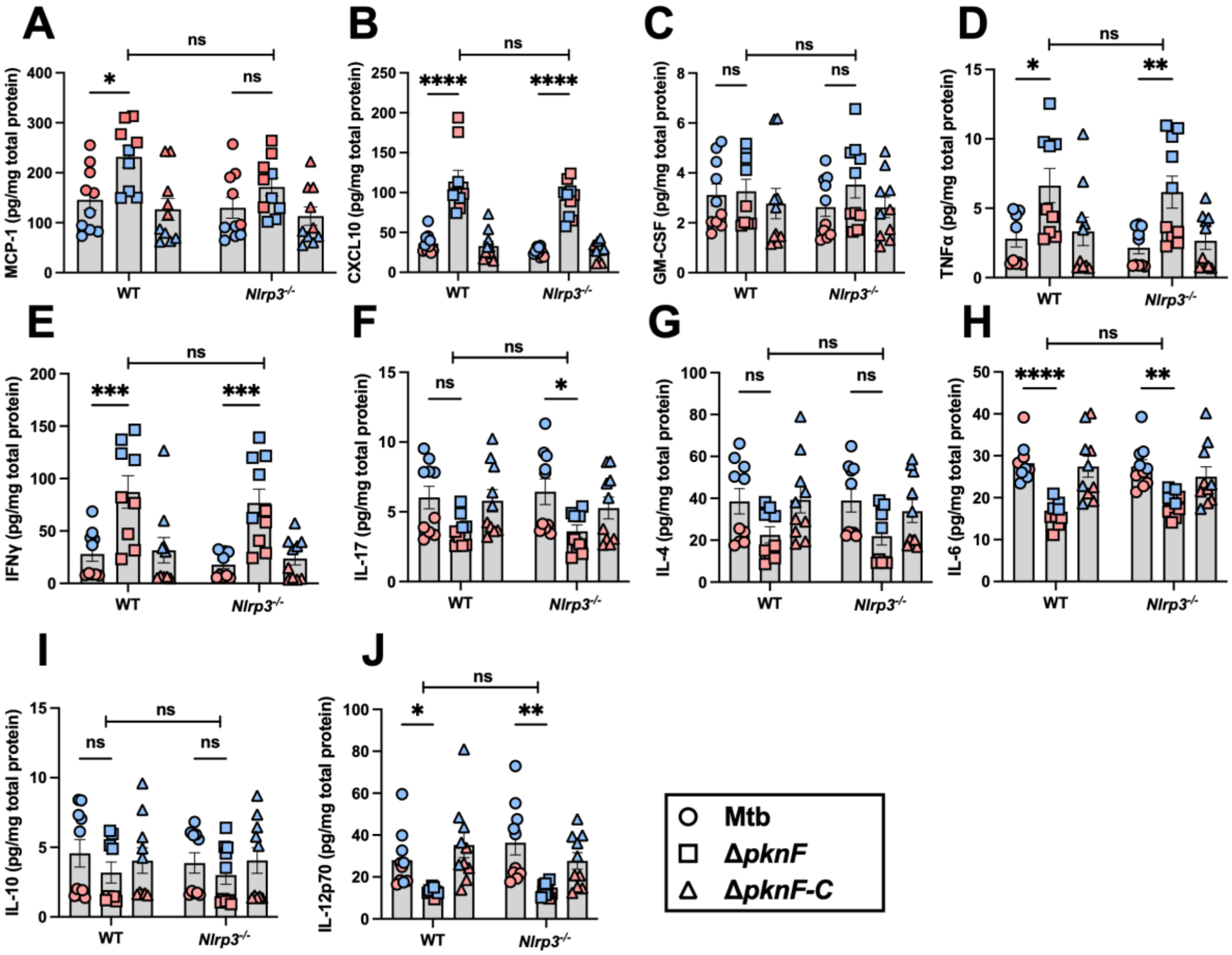

Supp. Fig. 3: Characterization of cytokine levels in lungs of wild-type and *Nlrp3*$^{-/-}$ mice at day 90-98 after Mtb infection.

Multiplexed ELISA (Luminex) was used to determine the amount of indicated cytokine normalized to total protein in the infected lungs described in Figure 3. Each point represents the result of one infected mouse from two independent infections (blue and pink colors). Normality of each data set was tested, and two-way ANOVA with Tukey's post-test was performed to compare experimental groups (* p<0.05, ** p<0.01, *** p<0.001, **** p<0.0001).
